# RACIAL/ETHNIC DISPARITIES IN TRAJECTORIES OF CARDIOVASCULAR RISK FACTORS IN US ADULTS

**DOI:** 10.1093/geroni/igae098.3299

**Published:** 2024-12-31

**Authors:** Radha Dhingra, Hanzhang Xu, Scott Lynch, Qing Yang, Michael Green, Jessica West, Matthew Dupre

**Affiliations:** Duke University, Durham, North Carolina, United States; Duke University, Durham, North Carolina, United States; Duke University, Durham, North Carolina, United States; Duke University, Durham, North Carolina, United States; Duke University, Durham, North Carolina, United States; Duke University, Durham, North Carolina, United States; Duke University, Durham, North Carolina, United States

## Abstract

Racial/ethnic disparities in major cardiovascular disease (CVD) risk factors are well documented. However, studies have not considered racial/ethnic differences in the interrelationships among these CVD risk factors and how they vary across age. We used six waves of longitudinal data from the Health and Retirement Study ([HRS] 2006-2016; n=10,292) to examine co-occurring changes in systolic blood pressure ([BP] mmHg), non-HDL cholesterol (mg/dl), diabetes (y/n), and smoking (y/n) at ages 50-80. Group-based multi-trajectory models were used to identify age-related trajectories of CVD risk factors and multinomial logistic regression models were used to identify participant characteristics associated with the trajectories of CVD risk factors. In our cohort (mean (SD) age: 62 (7.2) years; 41% male; 66% non-Hispanic White, 17% non-Hispanic Black, 13% Hispanic, and 3% other race/ethnicity), we found eight distinct trajectories of age-related changes in CVD risk factors (37% of adults had optimal CVD levels). We also found that non-Hispanic Black and Hispanic adults were significantly more likely to exhibit elevated age-related risks related to BP, cholesterol, diabetes, and/or smoking compared with non-Hispanic White adults. The associations were differentially attenuated with the inclusion of multiple covariates and showed that education, household income, and nativity status (among Hispanics) contributed most to the excess CVD risks observed in non-Hispanic Black and Hispanic adults. Overall, our findings demonstrate the impact of social determinants on racial/ethnic disparities in age-related changes in CVD risk factors in middle-aged and older adults in the United States.

